# Performance of the coronary calcium score in an outpatient chest pain clinic and strategies for risk stratification

**DOI:** 10.1002/clc.23611

**Published:** 2021-05-12

**Authors:** Weiting Huang, Cheney Jianlin Wong

**Affiliations:** ^1^ National Heart Centre Singapore Singapore Singapore

The intent of this workflow is to safely exclude coronary artery disease in chest pain clinics. Our risk scoring system showed similar performance to cardiac stress tests (sensitivity 89.3% and specificity 74.7%). In elevated risk patients (defined as risk >10% on Model 1 and Model 2), managing physicians may initiate guideline directed medical therapy, monitor their symptoms, keeping in view further sequential testing or coronary angiography should symptoms persist. The information gained from the CAC is also helpful in advising primary prevention.^1^

While chest pain history was important, feature importance analysis in our study showed that it fell behind the coronary calcium score in detecting coronary artery disease. Attention to classical symptoms may disadvantage women, the elderly, and individuals with diabetes, who may experience less‐typical symptoms.^2^, ^3^ An abstract our team published demonstrated that chest pain history did not significantly improve coronary artery disease prediction when the calcium score is considered.^4^

Additionally, from a standpoint of a specialist chest pain clinic, patients may present with unevaluated risk factors. This causes underestimation of coronary artery disease risk. The ideal workflow of having risk factors evaluated prior to the chest pain clinic is not always be feasible.^5^ Our study showed that even in the absence of risk factor status, the model still performed well in detecting CT‐demonstrated coronary artery stenosis, with sensitivity and specificity of 88.1% and 71.8%, respectively.

Our study provides an alternative workflow in evaluating coronary artery disease, acknowledging limitations in study design. This adds to the growing literature of utilizing calcium score in coronary artery disease patients.
